# *Nox*2 Deficiency Reduces Cartilage Damage and Ectopic Bone Formation in an Experimental Model for Osteoarthritis

**DOI:** 10.3390/antiox10111660

**Published:** 2021-10-22

**Authors:** Nik N. L. Kruisbergen, Irene Di Ceglie, Yvonne van Gemert, Birgitte Walgreen, Monique M. A. Helsen, Annet W. Slöetjes, Marije I. Koenders, Fons A. J. van de Loo, Johannes Roth, Thomas Vogl, Peter M. van der Kraan, Arjen B. Blom, Peter L. E. M. van Lent, Martijn H. J. van den Bosch

**Affiliations:** 1 Experimental Rheumatology, Department of Rheumatology, Radboud University Medical Center, 6525GA Nijmegen, The Netherlands; Nik.Kruisbergen@radboudumc.nl (N.N.L.K.); Irene.DiCeglie@radboudumc.nl (I.D.C.); Yvonne.vanGemert@radboudumc.nl (Y.v.G.); Birgitte.Walgreen@radboudumc.nl (B.W.); Monique.Helsen@radboudumc.nl (M.M.A.H.); Annet.Sloetjes@radboudumc.nl (A.W.S.); Marije.Koenders@radboudumc.nl (M.I.K.); Fons.vandeLoo@radboudumc.nl (F.A.J.v.d.L.); Peter.vanderKraan@radboudumc.nl (P.M.v.d.K.); Arjen.Blom@radboudumc.nl (A.B.B.); Martijn.vandenBosch@radboudumc.nl (M.H.J.v.d.B.); 2 Institute of Immunology, University of Münster, 48149 Muenster, Germany; rothj@uni-muenster.de (J.R.); vogl@uni-muenster.de (T.V.)

**Keywords:** osteoarthritis, ROS, Macrophages, NOX2, low-density lipoprotein

## Abstract

Osteoarthritis (OA) is a destructive disease of the joint with age and obesity being its most important risk factors. Around 50% of OA patients suffer from inflammation of the synovial joint capsule, which is characterized by increased abundance and activation of synovial macrophages that produce reactive oxygen species (ROS) via NADPH-oxidase 2 (NOX2). Both ROS and high blood levels of low-density lipoprotein (LDL) are implicated in OA pathophysiology, which may interact to form oxidized LDL (oxLDL) and thereby promote disease. Therefore, targeting NOX2 could be a viable treatment strategy for OA. Collagenase-induced OA (CiOA) was used to compare pathology between wild-type (WT) and Nox2 knockout (*Nox2^−/−^*) C57Bl/6 mice. Mice were either fed a standard diet or Western diet (WD) to study a possible interaction between NOX2-derived ROS and LDL. Synovial inflammation, cartilage damage and ectopic bone size were assessed on histology. Extracellular ROS production by macrophages was measured in vitro using the Amplex Red assay. *Nox2^−/−^* macrophages produced basal levels of ROS but were unable to increase ROS production in response to the alarmin S100A8 or the phorbol ester PMA. Interestingly, *Nox2* deficiency reduced cartilage damage, synovial lining thickness and ectopic bone size, whereas these disease parameters were not affected by WD-feeding. These results suggest that NOX2-derived ROS are involved in CiOA development.

## 1. Introduction

Osteoarthritis (OA) is a debilitating joint disease characterized by degradation of articular cartilage, formation of ectopic bone, and inflammation of the synovial membrane, which is the tissue that lines the joint cavity. The most important risk factors for OA include age, obesity, and previous joint injury. OA accounts for 2,4% percent of years lived with disability, while the global prevalence of knee and hip OA is approaching 5%, and without effective intervention will keep increasing as a result of an aging and advancingly obese population [1]. Current treatment options are limited to lifestyle interventions, analgesics and joint replacement at a far progressed disease state, urging for novel therapeutic options.

The classical view of OA as a mechanical ‘wear-and-tear’ process of the cartilage has changed in the last two decades towards a more complex disease of the whole joint. Many researchers now consider that chronic low-grade inflammation of the synovium co-mediates and accelerates OA development by causing an imbalance in both catabolic and anabolic processes [2]. Around 50% of OA patients suffer from synovitis, during which the number of macrophages increases which is accompanied by increased oxidative stress and production of reactive oxygen species (ROS) [3,4,5]. Increased ROS production contributes to disease via different pathways including deregulation of matrix synthesis and degradation and induction of chondrocyte apoptosis [6,7,8,9]. So far, general anti-oxidative therapies have failed to provide consistent beneficial effects on age related diseases such as OA [10,11,12,13]. However, many redox regulated cell signaling pathways are affected by these types of therapy, which may be causing undesired side-effects via some pathways, and thereby cover up the beneficial effects of interference in others. Inhibiting specific enzymatic sources of ROS may be a strategy to improve drug efficacy and minimize interference in homeostatic redox signaling pathways.

The major sites of ROS production include the mitochondria, where ROS are formed as a by-product of oxidative phosphorylation, and membrane bound nicotinamide adenine dinucleotide phosphate (NADPH) oxidase (NOX) complexes, which are especially expressed by phagocytes and are important in defense against pathogens. The NOX2 complex is mainly expressed by macrophages and polymorphonuclear neutrophils (PMNs) and is composed of transmembrane proteins NOX2 (or CYBB) and CYBA, cytosolic subunits NCF1 (organizer), NCF2 (activator), NCF4 and a GTPase. Assembly of the complex, including translocation of the cytosolic subunits to the cell membrane, is initiated after phosphorylation of NCF1 which leads to generation of superoxide (O_2_^•−^) in the extracellular milieu via the catalytic subunit NOX2 [14]. Inducers of NOX2 activity include cytokines, glucose intermediates and Toll-like receptor (TLR) ligands, such as the alarmin S100A8/A9. This protein complex is produced and released by activated macrophages and neutrophils in the OA synovium, which is reflected by elevated levels of S100A8/A9 in blood and synovium of OA patients, and has been shown to mediate Collagenase-induced OA (CiOA) development in mice [15,16].

Recent studies have suggested a disease-promoting role for NOX2 in OA; expression of NOX2 was increased in the synovial membrane of knee OA patients compared to controls which was associated to expression of NLR family pyrin domain containing 3 (NLRP3) [17]. Systemic factors such as high low-density lipoprotein (LDL) levels are associated with OA development and drive macrophage activation and ROS production [18,19]. NOX2-derived ROS might adopt an extra deleterious role in the context of high systemic levels of LDL as a result of oxidized LDL (oxLDL) formation, which has been implicated in ectopic bone formation [20,21,22]. In line with this, *Nox2* knockout and inhibition of NOX2 have shown beneficial effects in models of atherosclerosis, of which the pathology is driven by macrophage-mediated inflammation and oxLDL formation [23,24,25,26]. Here, we hypothesize that NOX2-derived ROS worsen OA pathology and that this deleterious effect can be aggravated when combined with high LDL levels as a result of western diet (WD) feeding.

## 2. Methods

### 2.1. Collection and Immunohistochemical Staining of Human OA Synovial Tissue

Synovial explants were obtained from anonymized leftover material of OA patients undergoing total knee replacement (*n* = 16). After 24 h of incubation at 37 °C in DMEM (+0.1% BSA) to obtain conditioned medium, tissue sections were fixed in formalin (4%) and embedded in paraffin. Histological sections were immunohistochemically stained using anti-CD68 (DakoCytomation M0814, 1:80) and anti-4-Hydroxynonenal (4-HNE) (R&D, Minneapolis, MN, USA: MAB3247, 0.02 µg/mL). Synovial explants from a second patient cohort undergoing total knee replacement (*n* = 22) were obtained and frozen in liquid nitrogen for later RNA isolation and quantitative polymerase chain reaction (qPCR) analysis. 

### 2.2. RNA Isolation of Human OA Synovial Tissue

Freshly isolated synovial tissue was snap frozen and preserved in liquid nitrogen. At the moment of RNA isolation, synovial tissue was transferred into MagNA Lyser green beads (Roche, Basel, Switzerland) and tissue samples were homogenized using the MagNA Lyser instrument (Roche, Basel, Switzerland). Total RNA was isolated using the RNeasy Fibrous Tissue Mini Kit (Qiagen, Hilden, Germany) according to the manufacturers protocol. RNA was treated with DNAse and reversed transcribed into complementary cDNA.

### 2.3. Animals

Wild type (WT) C57Bl6/J mice were obtained from Charles River, and *Nox2^−/−^* and Ncf1*^/^* mice (carrying a natural loss-of-function single nucleotide polymorphism in *Ncf1*) with a C57Bl6/J background were obtained from Jackson and subsequent in-house breeding. Female and male WT and *Nox2^−/−^* mice were 21 weeks old at the time of induction of the disease model, were housed in individually ventilated (IVD) cages and received food and water *ad libitum*. Cages were allocated to end-point day 7 or day 21 of CiOA and were fed either standard diet or WD (15% cacao butter, 1% cholesterol, 0.05% sodium cholate. Metabolized energy: 43% from fat, 42% from carbohydrates, 15% from protein (Sniff Spezialdiäten GmbH, Soest, Germany)), starting six weeks before induction of the disease model (switching back to standard diet temporarily from week −4 to −2 prior to induction (Figure 2A)) and continuing until the end of the experiment. Mice with dislocated joints were excluded from analysis, since dislocated joints develop extreme CiOA symptoms that are not anymore comparable to normal CiOA. Cage to experimental group allocation was done so that the age-spread and number of mice per cage were most optimally matched between experimental groups. Animal studies were approved by the Institutional Review Board and were performed according to the related codes of practice (CCD project number: 2018-0002).

### 2.4. Collagenase-Induced Osteoarthritis

Collagenase-induced OA (CiOA) was induced according to our current standard protocol, as previously published [27]. On day zero and day two of CiOA, mice received an intra-articular injection of 1U bacterial collagenase type VII (Sigma-Aldrich, St. Louis, MO, USA) into the right knee cavity. Investigators were aware of diet groups due to bright green food coloring in the WD, but blinded for genotype. On day 7 and day 21 of CiOA, mice were weighed, blood was collected via orbital plexus bleeding and mice were sacrificed, after which right knee joints were isolated and processed for histological analysis. On day seven of CiOA, the synovia were dissected from the knees before histological processing, weighed, and incubated for 2 h in DMEM (0.5% BSA) to obtain conditioned media. Synovial punches for qPCR analyses were obtained from a different experiment in which the identical diet was fed to C57Bl/6 mice starting four weeks prior to CiOA induction [27]. Serum samples were obtained from all mice to determine lipoprotein, S100A8/A9 and cytokine concentrations.

### 2.5. Amplex Red Hydrogen Peroxide Assay on PMNs and Macrophages

The Amplex Red reagent (ThermoFisher, Waltham, MA, USA) was used to measure hydrogen peroxide production in culture medium of live bone-marrow derived PMNs and macrophages. PMNs were derived from freshly isolated bone marrow cells at day 21 of CiOA using labelling with anti-Ly6G-Biotin and Streptavidin-Microbeads, after which Ly6G^+^ cells were isolated using MACS cell separation (Miltenyi Biotec, Bergisch Gladbach, Germany). Macrophages were derived from frozen bone-marrow cells of naïve mice (in 90% FCS + 10% DMSO) by culturing in DMEM (10% fetal calf serum (FCS), pyruvate, penicillin/streptomycin) in presence of rM-CSF (15 ng/mL) for six days at 37 °C. The Amplex Red reaction mixture was prepared in phenol-red free RPMI (1% FCS, 50 µM Amplex Red, 0.1 U/mL horseradish peroxidase (HRP)) and was added to washed cells in 96-well black/clear bottom plates and incubated at 37 °C before and in between measurements. Fluorescence was measured using a Clariostar microplate reader (excitation 545 nm, emission 590 nm). Extracellular hydrogen peroxide production was expressed as fluorescence intensity (FI) corrected for background (FI from Amplex Red reaction mixture without cells) and diphenyleneiodonium (DPI) (FI from Amplex Red reaction mixture on cells in presence of DPI (8 µM)).

### 2.6. Histological Analysis of Murine Knee Joints

Histological processing and analysis of murine knee joints was performed according to our current standard protocol, as previously published [27]. Isolated murine knee joints were fixed in 4% buffered formalin and subsequently decalcified in formic acid and embedded in paraffin. Synovial lining thickness was scored on H&E-stained sections using an arbitrary scoring system ranging from 0–3 (0 being no infiltration and 3 being the highest level of infiltration in this specific set of samples) (three sections per knee joint). Cartilage damage in the tibiofemoral joint was scored using a more detailed version of the OARSI score, in which the grade (severity of erosion scored 1–6) is multiplied by the stage (affected area scored 1–5), resulting in a score of 0–30 (five sections per knee joint) [28]. Osteophytes and enthesophytes were manually traced by an investigator, after which the surface area was calculated by Leica-software (three sections per knee joint). All histological analyses were performed blinded for the experimental condition. Both tibiofemoral and patellofemoral dislocations, as assessed on histology, were excluded posterior to scoring and prior to analysis.

### 2.7. Quantitative PCR

Gene expression levels were determined using qPCR with specific primers (primer sequences provided in Supplementary Figure S4) and the SYBR Green Master Mix using the StepOnePlus RT-PCR System (Thermo Fisher Scientific, Waltham, MA, USA). Expression levels are presented as −ΔCt, which is derived by correcting for the household gene glyceraldehyde-3-phosphate (GAPDH): Ct(GAPDH)—Ct(gene of interest).

### 2.8. Statistical Analysis

We based our sample size calculation on the hypothesis that there would be a significant interaction between the independent variables diet (standard diet or WD) and genotype (WT or *Nox2^−/−^*) in determining the level of the dependent variable cartilage damage. This experimental design was similar to one of our recently published studies and power analysis was therefore performed accordingly [27]. Group sizes were calculated to be able to detect differences between groups with a power of 0.8 and a level of significance of 0.05, using Russ Lenth’s sample size calculator (version 1.76) for the primary readout measure cartilage damage tested with a two-way ANOVA, considering a difference smaller than 3.2 as not biologically relevant. Assuming a mean of 10 in the control group (female C57Bl/6 mice, standard diet, untreated), a standard deviation of 2 and a detectable contrast of 3.2, this resulted in a total required number of 10 mice per group. Due to a small breeding deficit, the two groups of female *Nox2^−/−^* mice ended up with eight and nine mice. Two-way ANOVA was used to test for interaction between diet (WD) and genotype (*Nox2^−/−^*) for all histological outcome parameters and qPCR, separately on both timepoints. One-way ANOVA with Dunnett’s post-test (compared to NS) was used to test for significant effects of stimuli in the Amplex Red assay, and Bonferroni’s post-test was used to test for effect of WD on lipoprotein levels. All statistical analyses were performed using Graphpad Prism v5.01. Pearson’s test was used to test for correlations, after D’Agostino and Pearson omnibus was used to test for normality. *p*-values lower than 0.05 were considered significant. Results are expressed as mean and standard deviation (SD) or mean and 95% Confidence Intervals (95% CI’s).

## 3. Results

### 3.1. Macrophage Marker CD68 and Oxidation Marker 4HNE Are Particularly Present in the Lining of Human OA Synovium, and CD163 Positively Correlates with NOX2

**Figure 1:**
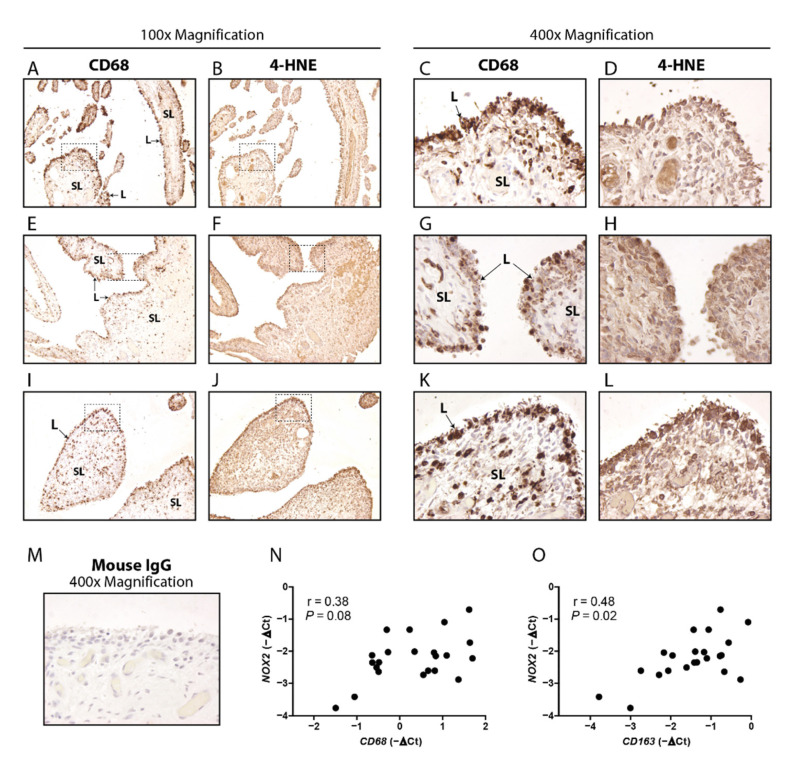
Macrophage marker *CD68* and oxidation marker *4HNE* are particularly present in the lining of human OA synovium, and *CD163* positively correlates with *NOX2*. Immunohistochemical staining of CD68 (**A**,**C**,**E**,**G**,**I**,**K**) and 4HNE (**B**,**D**,**F**,**H**,**J**,**L**) was performed on sections of human OA synovium (100× and 400× magnification). Adjacent sections from synovium of three different patients are shown (**A**–**L**). Dashed square shows area of 400× magnification. L = lining, SL = sub-lining. Mouse IgG was used as isotype control (**M**). mRNA expression of *NOX2* did not correlate with *CD68* (**N**), but did positively correlate with *CD163* (**O**) in human OA synovium.

First, we determined the abundance of macrophages, the extent of lipid peroxidation and their colocalization in human end-stage OA synovium by conducting immunohistochemistry for pan-macrophage marker CD68 and lipid peroxidation marker 4HNE respectively. CD68 positive cells were clearly distinguishable and seen in both synovial lining and sub-lining, but mostly in the lining (Figure 1A,C,E,G,I,K, sections from three different patients). 4HNE was observed throughout the tissue while the most intense staining was also located in the synovial lining (Figure 1B,D,F,H,J,L, sections from three different patients, adjacent to those shown in Figure 1A,C,E,G,I,K). Expression of *NOX2,* the gene encoding NOX2 protein, was not significantly correlated with *CD68* expression as analyzed with qPCR (r = 0.38, *p* = 0.08) (Figure 1N). Interestingly, however, *NOX2* did show a positive correlation with *CD163*, a marker for alternatively activated macrophages (r = 0.48, *p* = 0.02) (Figure 1O).

### 3.2. WD-Feeding Increased Serum LDL Levels

**Figure 2:**
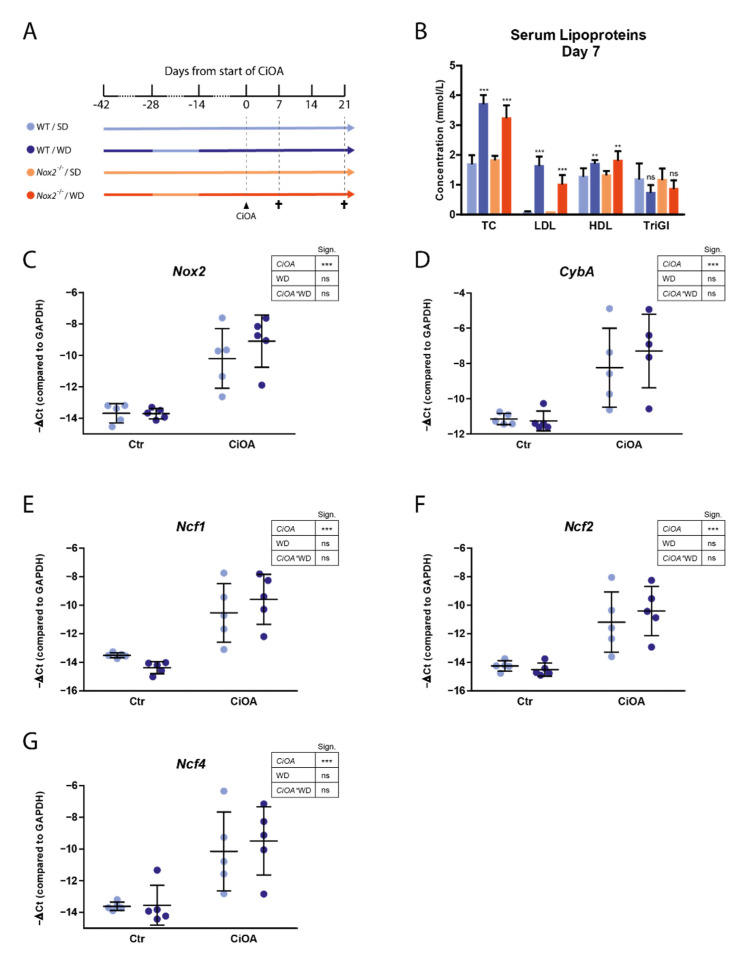
WD-feeding increases serum LDL levels and CiOA induction increases expression of NOX2-complex genes in mouse synovium. (**A**) Schematic of experiment design. C57Bl/6 and *Nox2^−/−^* mice were fed either a standard diet (SD) or western diet (WD). Mice were sacrificed on day 7 and 21 of CiOA. *n* = 6 per group for day 7 and *n* = 10 per group for day 21. (**B**) Serum concentrations of total cholesterol (TC), low-density lipoprotein (LDL), high-density lipoprotein (HDL) and triglycerides (TriGl) were determined at day 7 of CiOA (*n* = 6). Significance levels show results of WD versus SD in each genotype separately. (**C**–**G**) mRNA expression of NOX2-complex genes *Nox2*, *Cyba*, *Ncf1*, *Ncf2* and *Ncf4* is increased in CiOA knee joints compared to contralateral control knee joints at day 7 of CiOA (*n* = 5). Data are presented as mean and SD (**B**) or mean and 95% CI (**C**–**G**). *p*-value summary resulting from two-way ANOVA shown in tables: ***, *p* < 0.001; **, *p* < 0.01; ns, *p* > 0.05.

Since a high number of male mice (14 out of 40) had to be excluded from analysis due to dislocated joints (Supplementary Figure S2A), we will focus on the results of the female mice in the rest of this manuscript. Seven weeks after initial start of Western diet feeding and seven days after CiOA induction, serum LDL levels were significantly enhanced in WD-fed mice compared to mice fed a standard diet in both WT and *Nox2* knockout (*Nox2^−/−^*) mice (1.55 and 0.94 mmol/L mean increase respectively) (Figure 2A,B). WD-feeding did not lead to an increase in weight in WT mice. *Nox2^−/−^* mice that were fed a WD showed a lower weight compared to the other groups. However, this was attributable to pre-existing group differences already present at the onset of WD-feeding (Supplementary Figure S1).

### 3.3. CiOA Induces Nox2 Upregulation in the Synovium at Early Stage CiOA

CiOA pathology is characterized by inflammation of the synovium especially at early stage, which is involved in driving cartilage destruction, bone pathology and ligament pathology at end stage pathology. To investigate changes in expression of NOX2-complex genes in the synovium at early stage CiOA, and the effect of WD-feeding on these changes, we performed qPCR analysis of RNA isolated from synovial punches. At day seven of CiOA, expression of *Nox2*, *Cyba*, *Ncf1*, *Ncf2* and *Ncf4* was increased in CiOA synovium compared to control synovium (17, 11, 15, 11 and 14-fold, respectively, *p* < 0.001 for all) (Figure 2C–G). WD-feeding did not affect expression of NOX2-complex genes.

### 3.4. Nox2^−/−^ Macrophages Produce Basal Levels of ROS but Fail to Upregulate These upon Stimulation with PMA and S100A8

**Figure 3:**
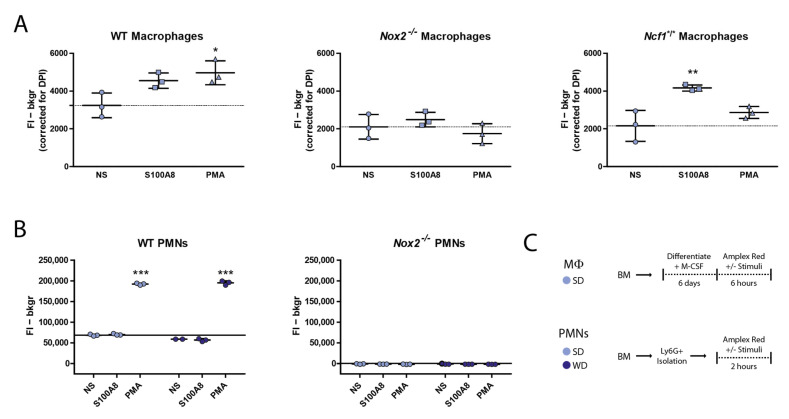
*Nox2^−/−^* macrophages produce basal levels of ROS but fail to upregulate these upon stimulation with PMA and S100A8. (**A**) Amplex Red assay readout (@545-15nm) of WT, *Nox2^−/−^* and Ncf1*^/^* macrophages differentiated for six days in presence of M-CSF (15 ng/mL) (**C**), and of WT an *Nox2^−/−^* PMNs (**B**) isolated from fresh bone marrow using positive selection of Ly6G^+^ cells (**C**). Data are presented as mean and SD. *, *p* < 0.05; **, *p* < 0.01; ***, *p* < 0.001 compared to non-stimulated (NS).

To confirm that *Nox2^−/−^* phagocytes are compromised in their ability to produce extracellular ROS, we performed an Amplex Red assay using WT and *Nox2^−/−^* macrophages. We included Ncf1*^/^* macrophages and both WT and *Nox2^−/−^* PMNs as controls (Figure 3A–C). In a previous study, we found that while Ncf1*^/^* PMNs were incapable of producing NOX-derived ROS, Ncf1*^/^* macrophages unexpectedly provided a positive Amplex Red signal in presence of S100A8 [29]. Here, we could repeat that Ncf1*^/^* macrophages indeed produced ROS in the presence of the relevant alarmin S100A8, whereas *Nox2^−/−^* macrophages failed to increase their ROS production under these circumstances (1.9-fold and 1.2-fold) (Figure 3A). Both genotypes did not increase ROS production upon PMA stimulation, whereas WT macrophages did (1.5-Fold). WT PMNs also showed a positive signal which was increased with PMA stimulation (2.8-Fold) (Figure 3B). In line with our expectations, *Nox2^−/−^* PMNs did not show any signal above background, also not in presence of PMA.

### 3.5. Nox2 Knockout Does Not Affect Synovial Lining Thickness and S100A8/A9 Expression at Day 7 of CiOA

**Figure 4:**
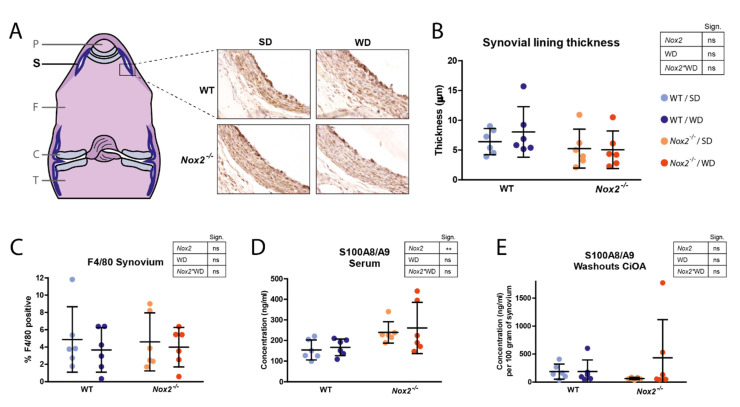
*Nox2* knockout does not affect synovial lining thickness and S100A8/A9 expression at day seven of CiOA. (**A**) Illustration shows a schematic overview of a frontal section of an H&E-stained knee joint. P, patella; S, synovium; F, femur; C, cartilage; T, tibia. Pictures show sections immunohistochemically stained for F4/80 and are representative of the mean of each group (400× magnification). (**B**) Synovial lining thickness at day seven of CiOA was scored on F4/80-stained histological sections by measuring the thickness in µm. Three sections of different standardized depths were scored per knee joint. (**C**) F4/80 positive staining was measured and quantified as the percentage of positive staining in the outer 200 µm of synovium. (**D**,**E**) Concentrations of S100A8/A9 in serum (**D**) and synovial washouts (**E**) using ELISA. Data are presented as mean and 95% CI’s. *n* = 10. *p*-value summary resulting from two-way ANOVA shown in tables: **, *p* < 0.01; ns, *p* > 0.05. No mice were excluded from analysis.

During synovitis development in OA, the number of macrophages in the synovium increases, which can be observed on histology as thickening of the synovial lining layer. Histological analyses of knee joints at day seven of CiOA did not reveal any significant effects of *Nox2* deficiency or WD-feeding on synovial lining thickness (Figure 4A,B). Also the percentage of synovium area positive for macrophage marker F4/80 was not significantly different between groups despite a trend towards a lower percentage in WD-fed mice (Figure 4A,C). Despite enhanced S100A8/A9 serum protein levels in *Nox2^−/−^* mice compared to WT mice (Figure 4D), concentrations in synovial washouts were not different (Figure 4E). Concentrations of cytokines in synovial washouts measured with Luminex, including IL-1β, IL-6, and TNF-α, were undetectable (data not shown).

### 3.6. Decreased Cartilage Damage and Ectopic Bone Formation in Nox2^−/−^ Mice at Day 21 of CiOA

**Figure 5:**
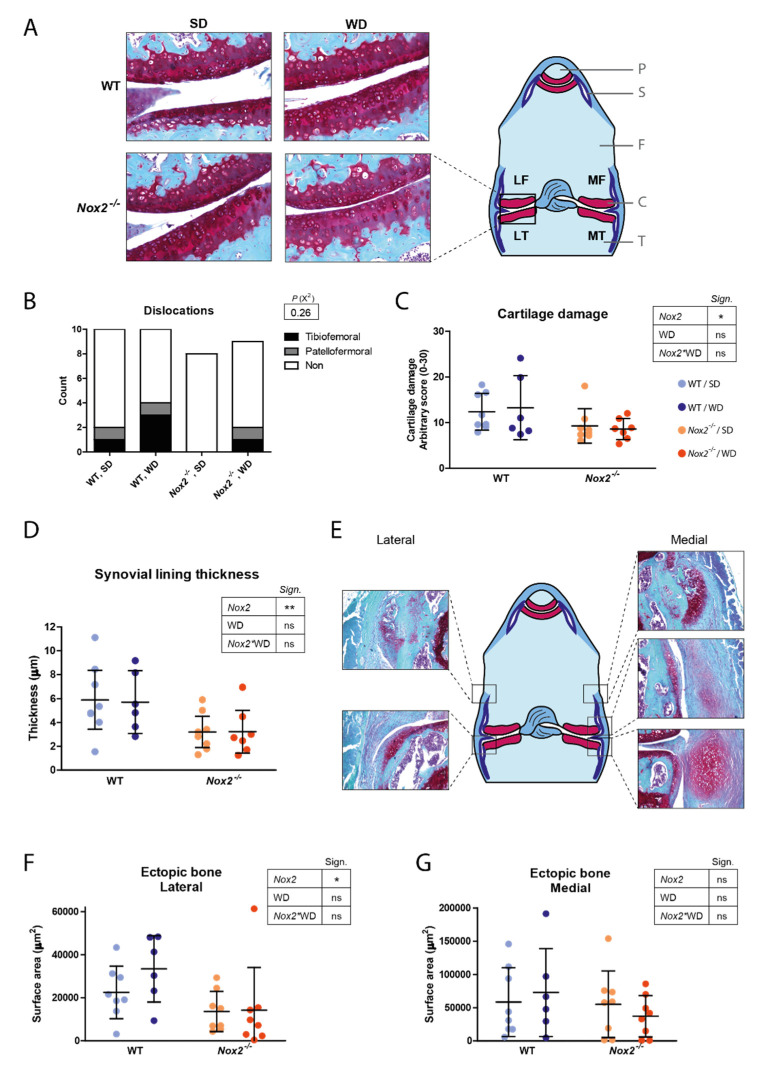
Decreased cartilage damage, synovial lining thickness and ectopic bone formation in *Nox2^−/−^* mice at day 21 of CiOA. (**A**,**E**) Illustrations each show a schematic overview of a frontal section of a SafO stained knee joint. P, patella; S, synovium; F, femur; C, cartilage; T, tibia. (**A**) Sites where cartilage was scored is presented as LF (lateral femur), MF (medial femur), LT (lateral tibia) and LF (lateral femur). Pictures are representative of the mean across groups (200× magnification). Dislocations were excluded from further analysis (**B**). (**C**) Cartilage damage in the tibial-femoral joint was scored using a modified OARSI score, in which the grade (severity of erosion) is multiplied by the stage (affected area), resulting in a score of 0–30 (0 = no damage, 30 = maximal damage). Five sections were scored per knee joint. (**D**) Synovial lining thickness at day 21 of CiOA was scored on H&E stained histological sections by measuring the thickness in µm. Three sections of different standardized depths were scored per knee joint. (**E**–**G**) Ectopic bone was scored at five different sites in the joint (**F**). Ectopic bone masses were manually circled by an investigator blinded for the experimental condition, after which the surface areas were calculated. The mean cross-sectional surface area in three sections per knee joint was determined, and the measurements on the lateral (**F**) and medial (**G**) side of the joint were averaged separately. Pictures are representative of the mean across groups (50× magnification). Data are presented as mean and 95% CI’s. *p*-value summary resulting from two-way ANOVA shown in tables: *, *p* < 0.05; **, *p* < 0.01; ns, *p* > 0.05.

Finally, we used histological sections of day 21 to assess whether *Nox2* deficiency reduced the degenerative disease manifestations typical of end-stage OA; cartilage damage and ectopic bone formation. Crucially, *Nox2* deficiency had a protective effect on structural cartilage damage compared to WT mice. This was shown by a 30% reduction in cartilage damage score in *Nox2^−/−^* mice compared to WT mice (*p* = 0.028) (Figure 5A,C). Whereas synovial lining thickness was not found to be affected by *Nox2* deficiency at day 7 of CiOA, it was significantly reduced at day 21 (44% reduction compared to WT, *p* = 0.006). In addition to cartilage damage and synovitis, the incidence of ectopic bone masses was visibly reduced in the lateral side of the knee joint of *Nox2^−/−^* mice compared to WT mice (Supplementary Figure S3). Also the average surface area of ectopic bone was decreased in *Nox2^−/−^* mice compared to WT mice at the lateral side of the knee joint (50% reduction compared to WT, *p* = 0.015) (Figure 5E–G). Although WD-feeding did not have an effect on cartilage damage and ectopic bone formation (*p* = 0.22), the number of dislocations (which were excluded from further analyses) were higher in WD-fed mice in both female and male mice, especially in WT mice (*p* = 0.26 in female and *p* = 0.04 in male mice) (Figure 5B and Supplementary Figure S1A).

## 4. Discussion

OA shares many risk factors and is associated with atherosclerosis (ATH), which makes it plausible that their underlying pathophysiology shares common biochemical pathways [30]. ATH development is dependent on local macrophage-mediated inflammation of the blood vessel wall accompanied with ROS production. Since macrophages are also strongly involved in OA pathology, NOX2-mediated ROS production in the synovium might be a disease-promoting process in a subtype of OA characterized by synovial inflammation. It was hypothesized that this effect of NOX2 on disease is aggravated in combination with high systemic levels of LDL as a consequence of oxLDL formation, which is an event that also occurs during atherosclerosis development. In line with this theory, in vivo studies using hypercholesterolemic mouse models showed aggravated OA pathology; *Ldlr^−/−^* mice, *ApoE^−/−^* mice, and WT mice fed a WD all showed increased ectopic bone formation during CiOA development [20,21,22]. In addition, surgically induced models of OA such as destabilization of the medial meniscus (DMM), as well as spontaneous OA, have shown increased cartilage damage in hypercholesterolemic mice [31,32,33].

Interestingly, we found that *Nox2* deficiency significantly decreased cartilage damage and ectopic bone formation compared to WT mice independent of WD feeding, indicating that NOX2-derived ROS mediate severity of CiOA pathology. In vitro studies using human chondrocytes have shown that ROS are involved in OA promoting processes including production of cytokines and matrix degrading enzymes [3]. Furthermore, NOX2-mediated ROS production led to increased expression of inflammatory mediators in rat chondrocytes [34]. To our knowledge however, this is the first time that beneficial effects of decreased NOX2-derived ROS have been reported in an in vivo OA model. NOX2-derived ROS can be involved in cartilage damage and ectopic bone via different mechanisms. ROS are implicated in chondrocyte hypertrophy, apoptosis and senescence, processes that precede cartilage damage during OA development. Lower levels of NOX2-derived ROS might have slowed down these deleterious processes in *Nox2^−/−^* mice. In addition, ROS activate latent enzymes and growth factors involved in OA pathology, most importantly matrix metalloproteinases (MMPs) and transforming growth-factor β (TGF-β), and could thereby mediate cartilage breakdown and ectopic bone formation respectively [35,36]. Furthermore, NOX2 has been implicated in bone remodeling by enhancing osteoclastogenesis, which is an important process during ectopic bone formation [37,38].

Interestingly, the reductions in cartilage damage and ectopic bone formation were accommodated with reduced synovial lining thickness at day 21 of CiOA. This is in line with the idea that synovitis co-determines the extent of cartilage damage and ectopic bone formation during OA development. Given that no effects on synovitis were found at day 7 of CiOA, this suggests that inflammation resolves faster during CiOA when NOX2-derived ROS are absent. In addition to readouts related to macrophage abundance, it might also be interesting to further investigate the effects of *Nox2* deficiency on macrophage phenotype and activation status. Indeed, NOX2 has been shown to be essential for macrophage differentiation in mice, especially for polarization to the M2-type [39].

The involvement of NOX2 in CiOA underlines the importance of macrophages in OA, thought to be the main NOX2-expressing cell in OA synovium. Staining of lipid peroxidation marker 4HNE seemed most intense in the lining of OA synovium, which is more macrophage-dense compared to the sub-lining. The 4HNE staining did not seem exclusive for a specific cell type but was instead more widespread. This could be explained by lipid peroxidation of cell membranes, which might also occur on cells that do not produce NOX2-derived ROS themselves. Furthermore, we show here that expression of *NOX2* is positively correlated with M2-macrophage marker *CD163* in human OA synovium. In combination with *NOX2* not correlating with pan-macrophage marker *CD68*, this could imply that in OA synovium, *NOX2* is mainly expressed by M2-like macrophages, which is considered more anti-inflammatory compared to other subtypes. Further investigation using more precise, single-cell based readouts is required to confirm this. 

NOX2 has repeatedly demonstrated to play a suppressive role in mouse models of rheumatoid arthritis (RA). RA is an autoimmune disease, and naturally occurring deficiencies in the *Ncf1* locus causing a reduced oxidative burst led to enhanced autoimmunity and arthritis [40,41]. The discrepancies between the role of NOX2 in OA and RA might be explained by the different types of immune cells important in their pathophysiology. Presence of PMNs is more pronounced in RA synovitis compared to OA synovitis, and is considered to be more involved in pathology. Activated PMNs have a short life span partly due to the high levels of ROS they produce themselves into the extracellular milieu via NOX2. When this self-regulating mechanism is not functional, PMN clusters are allowed to expand to form granuloma’s as is the case in chronic granulomatous disease, which is strongly associated to NOX2 associated gene deficiencies [42]. Therefore, NOX2 deficiency could worsen PMN-mediated pathology, such as RA, whereas pathology mediated by other cell types remain unaffected or are ameliorated. In this study, CiOA was not aggravated but ameliorated, and no granulomas were observed. This seems to be in line with the idea that PMNs do not play an important role in CiOA development.

The significant effect of *Nox2* knockout on pathology was not anticipated without the combination with WD since we previously showed that *Ncf1* deficiency did not affect CiOA pathology in mice [29]. When considering effect size, Ncf1*^/^* mice did show a trend in reduced cartilage damage of about 10% compared to WT mice but this was too low to be clinically relevant. In addition, this is significantly lower than the effect seen in *Nox2^−/−^* mice, which showed a 30% reduction in cartilage damage score compared to WT mice. These differences in outcome may be explained using the finding that Ncf1*^/^* macrophages were shown to still produce ROS in presence of S100A8. Although *Ncf1* knockouts are often assumed to be functional knockouts of the entire NOX2 complex, this might become undone in an environment with S100A8/A9 presence such as a CiOA joint. Confirming this, studies have shown that S100A8/A9 can directly bind to p67phox to activate the NOX2 complex [43,44,45]. Taking this into consideration, we think *Nox2^−/−^* is a more suitable model to investigate the role of NOX2 in pathologies with involvement of macrophages. When using an Ncf1*^/^* model, disease development will be mediated by macrophages still able to produce superoxide via the NOX2-complex in an NCF1-independent (and possibly S100A8/A9-dependent) manner.

In contrast to beforementioned studies [20,21], WD feeding did not increase CiOA pathology in the present study despite increased serum LDL levels. This is in line with a recently published study of our group in which an identical WD was used [27]. The difference in outcomes of these two studies compared to the ones mentioned in the previous paragraph could possibly be explained by presence of sodium cholate (0.05%) in the specific diet used here, which was not supplemented to WDs used in previous experiments. Addition of this bile acid makes WD especially effective in enhancing serum LDL levels but it might have additional side effects which may counteract the OA aggravating effects of WD, for example via its proven ability to locally suppress innate immunity [27,46]. The latter might be especially relevant for OA given the central role that innate immunity is thought to play in its pathophysiology. To evaluate whether the WD-aggravated part of CiOA pathology is indeed dependent on NOX2-derived ROS production, it would be interesting to repeat the experiment using a different diet or genetic background so that the aggravating effects of WD previously found are recreated.

In addition to the effects found on cartilage damage, synovitis and ectopic bone formation, a high number of total dislocations were found in male mice (14 out of 40 mice), which were disproportionally higher in WD-fed WT mice compared to the other three groups (7 out of 14 dislocations) (Supplementary Figure S2A). The dislocations in the female mice showed a similar pattern, although with a lower incidence (Figure 5B). The intra-articular injections of collagenase into the knee joint causes damage to collagen type I containing structures such as tendons, menisci, and ligaments leading to destabilization of the joint, which in severe cases can cause a complete dislocation [47]. WD-feeding seems to increase the number of dislocations, particularly in WT mice compared to *Nox2^−/−^* mice, suggesting that a WD-associated factor and NOX2-derived ROS interact to increase collagenase-induced destabilization of the joint.

The interpretation of the study results are subject to some limitations. One of the experimental groups (WT, WD-fed) coincidentally had a slightly deviating average weight at the start of the experiment. Although we therefore can’t exclude the possibility that weight differences affected the outcome, the differences in outcome parameters are also seen between the groups of standard diet fed mice, which do not differ in weight, which makes it unlikely that the differences in pathology between WT and *Nox2^−/−^* mice are mainly weight mediated. Furthermore, we believe an optimal study design would include equal analysis of both female and male mice to identify sex differences. However, and unfortunately, we had to exclude analysis of males due to the high number of dislocations.

## 5. Conclusions

Here, we show that *Nox2* deficiency reduces cartilage damage, synovial lining thickness and ectopic bone formation during CiOA in female mice. This suggests that inhibiting NOX2 during OA development could be a viable therapeutic option to relieve these disease symptoms. Efficacy of specific NOX2 inhibitors which have shown promising results in studies on atherosclerosis and spinal cord injury (such as gp91ds-tat) could be further investigated for OA [48,49].

## Data Availability

The data presented in this study are available in article.

## Supplementary Materials

The following are available online at https://www.mdpi.com/article/10.3390/antiox10111660/s1, Figure S1: Body weight of female mice, Figure S2: Dislocations and cartilage damage in male mice, Figure S3: Ectopic bone incidence in female mice, Figure S4: qPCR primer sequences.
