# Supplementary figures and images for: Nox2 Deficiency Reduces Cartilage Damage and Ectopic Bone Formation in an Experimental Model for Osteoarthritis
Source: Antioxidants (Basel). 2021 Oct 22;10(11):1660. doi: 10.3390/antiox10111660 (PMC8614813; doi:10.3390/antiox10111660)

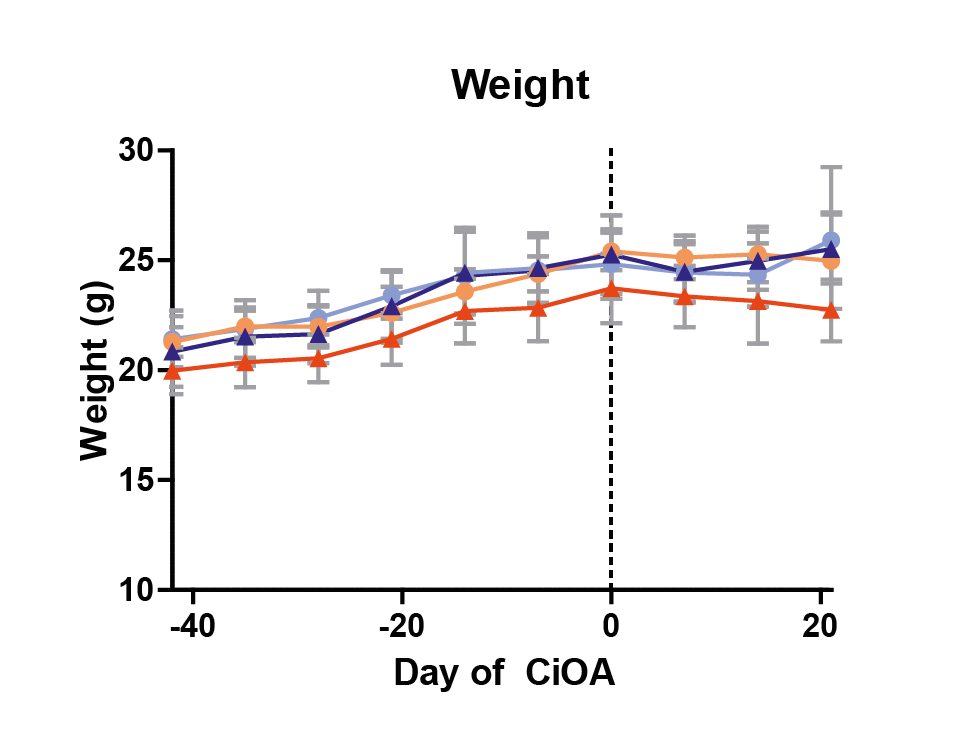

Supplement: Supplementary file 1 [file antioxidants-10-01660-s001.zip › NK4 supplementary Figure S1.png]

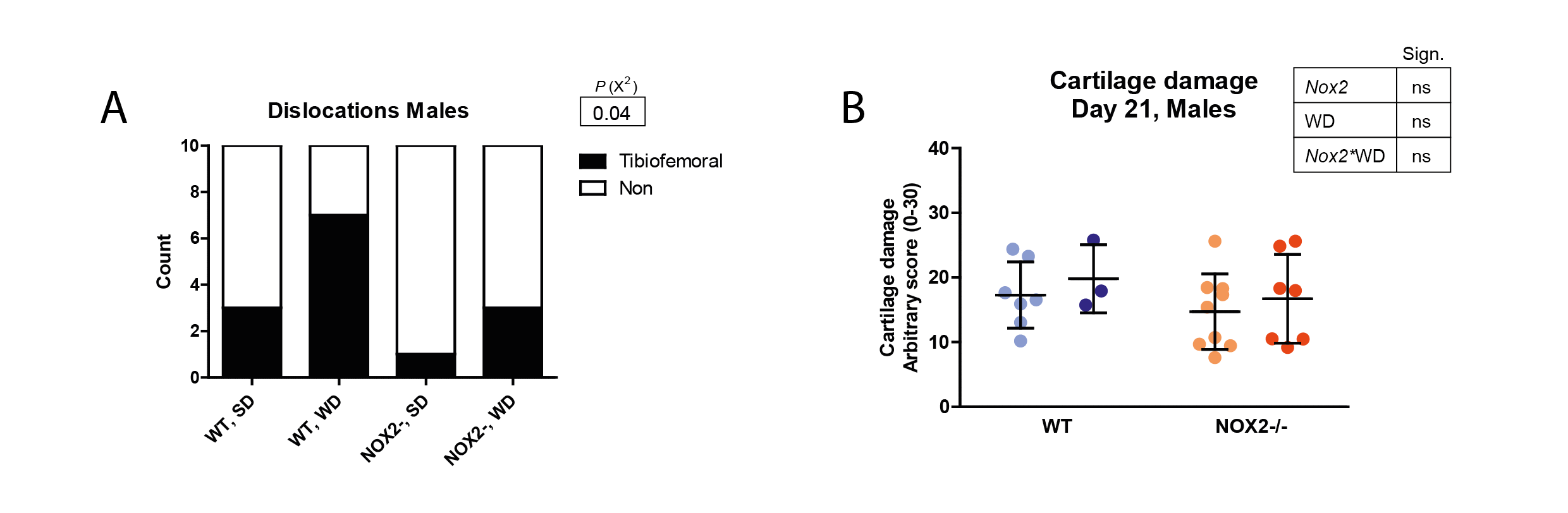

Supplement: Supplementary file 1 [file antioxidants-10-01660-s001.zip › NK4 supplementary Figure S2.png]

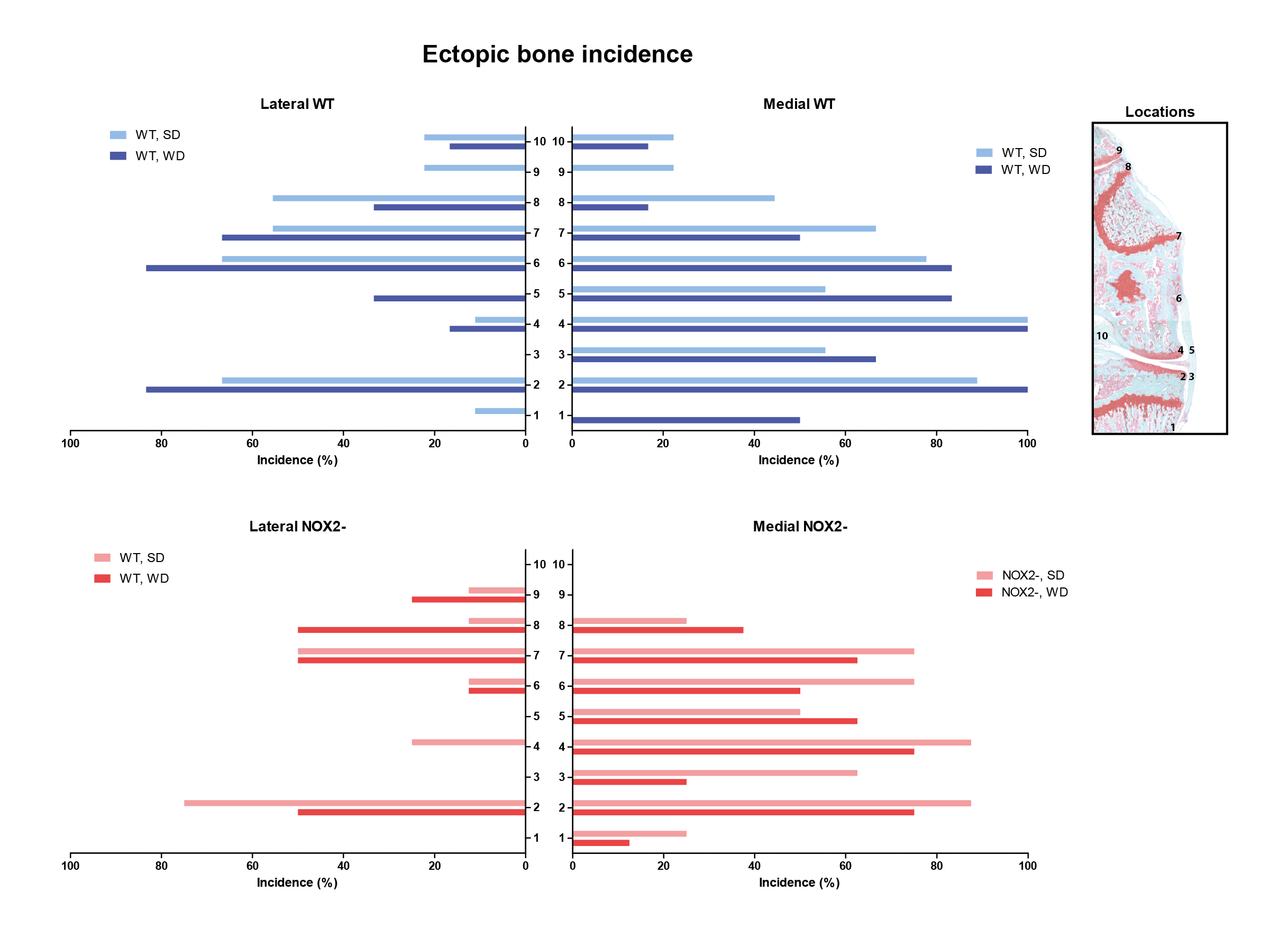

Supplement: Supplementary file 1 [file antioxidants-10-01660-s001.zip › NK4 supplementary Figure S3.png]

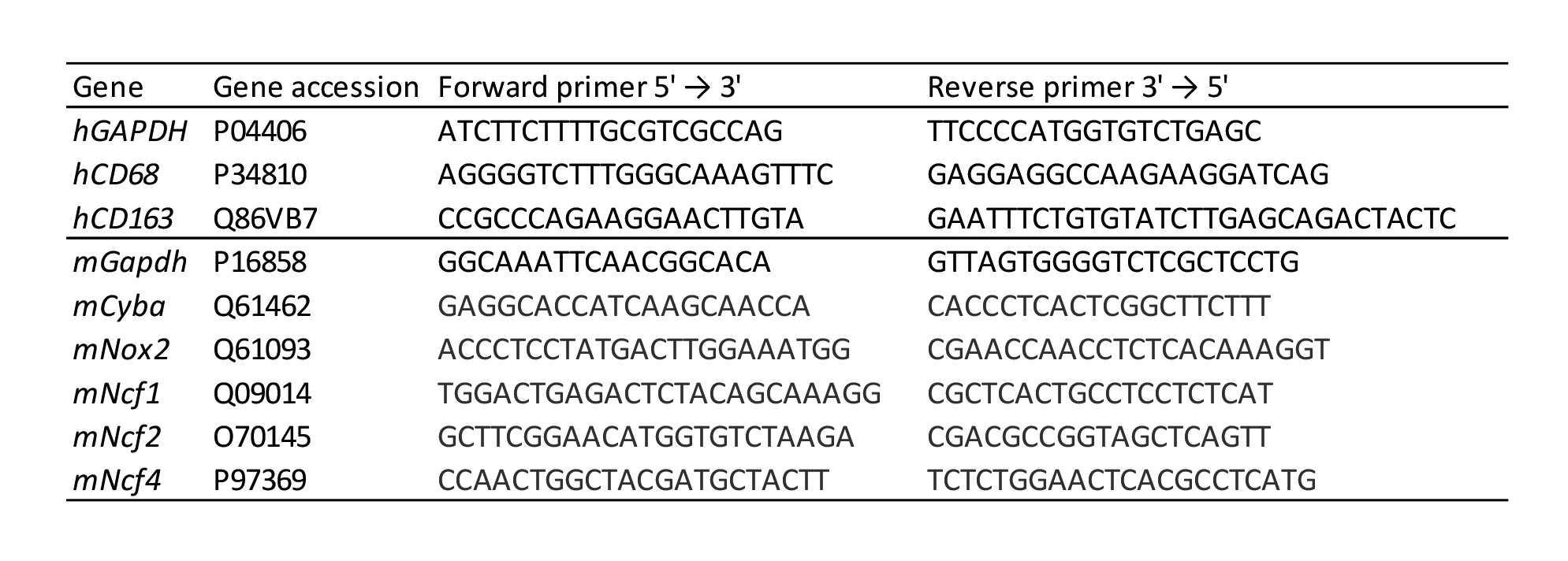

Supplement: Supplementary file 1 [file antioxidants-10-01660-s001.zip › NK4 supplementary Figure S4.png]
